# Towards Antimicrobial Formulations of Repurposed Drugs and Cationic Surfactants: Efficacy, Cytotoxicity on Human Cell Lines, and Ecotoxicity in Aquatic Models

**DOI:** 10.3390/antibiotics15080721

**Published:** 2026-07-24

**Authors:** Alida Monreal, Diego Ballestero, Cristina Yus, Iman Mattar, Gracia Mendoza, Elisa Langa, Mª Rosa Pino-Otín, Manuel Arruebo

**Affiliations:** 1 Instituto de Nanociencia y Materiales de Aragón (INMA), CSIC-Universidad de Zaragoza, 50009 Zaragoza, Spain; 2 Aragon Health Research Institute (IIS Aragon), 50009 Zaragoza, Spain; 3 Universidad San Jorge, Villanueva de Gállego, 50830 Zaragoza, Spain; 4 Department of Chemical and Environmental Engineering, University of Zaragoza, Campus Río Ebro-Edificio I+D, C/Poeta Mariano Esquillor S/N, 50018 Zaragoza, Spain

**Keywords:** antimicrobial combination therapy, drug repurposing, intracellular infection, 3D cell culture models, ecotoxicity

## Abstract

Background: Antimicrobial combination therapies outperform traditional monotherapies by offering additive or synergetic effects, a broader spectrum of activity, enhanced efficacy against polymicrobial infections, and a reduced risk of resistance development and infection relapse. Methods: Herein, we have combined the chemotherapeutic antibiotic alkylating agent mitomycin C (MMC) with the wide-spectrum antiseptic octenidine hydrochloride (OCT). The non-specific mode of action via membrane disruption of the latter, together with the DNA-alkylating ability of the former, is here combined to produce bactericidal antibiotic-like mixtures against clinical isolates of Methicillin-resistant *Staphylococcus aureus* and uropathogenic *Escherichia coli*. The concentrations required for both antimicrobials to inhibit or eradicate bacterial biofilms as well as intracellular persisters are also determined here. The cytotoxicity of the selected combinations on eukaryotic cells was also evaluated in traditional 2D cultures and in 3D-fibroblast spheroids to mimic a physiologically relevant microenvironment present in topical infections. Finally, the ecotoxicity of the studied compounds was also evaluated through standardized bioassays using representative microorganisms commonly employed in environmental toxicity assessments such as *Daphnia magna* (*D. magna*) and *Aliivibrio fischeri* (*A. fischeri*). Results: Compared to monotherapy, these environmentally friendly antibiotic-like combinations at specific concentrations may achieve additive antimicrobial effects against planktonic, sessile, and intracellular pathogenic strains. However, the ecotoxicological response was bioindicator-dependent: the OCT–MMC combination reduced toxicity in *A. fischeri*, but not in *D. magna*, where toxicity was comparable to OCT alone, indicating OCT as the main driver. Thus, any reduction in ecotoxicity appears species-specific. Nonetheless, the use of lower doses may help minimize selective pressure for resistance. Conclusions: Collectively, these findings demonstrate that selected MMC–OCT combinations achieve antibacterial efficacy against planktonic, sessile, and intracellular pathogenic bacteria while preserving the viability of eukaryotic cells and exhibiting minimal toxicity toward standard ecotoxicological model organisms. Further studies are needed to guarantee the safe use of this combination at selected doses and to guarantee its non-systemic absorption after topical administration.

## 1. Introduction

Mitomycin C (MMC) is a chemotherapeutic antibiotic used in the treatment of gastrointestinal and urogenital cancers [1]. The aziridine group present in MMC acts as an alkylating agent, crosslinking the DNA complementary strands where cytosine is followed by guanine along its 5′→3′ direction (i.e., CpG region), inhibiting DNA replication and transcription [2]. Despite its bactericidal and viricidal character at high doses, its antibiotic use is restricted due to its high cytotoxicity. However, high antimicrobial action has been demonstrated for MMC against planktonic, sessile, and even intracellular persisters (i.e., small colony variants) following a growth-independent mechanism [3]. MMC is also able to eliminate both Gram-positive and Gram-negative bacteria in mono- and co-cultures of bacterial biofilms in vitro and in vivo [3].

At subcytotoxic doses, repurposed MMC has been used in combination with commercial antibiotics to reduce the minimum bactericidal doses of the latter and consequently to potentially reduce the chances of genetic selection to develop resistance. For instance, synergetic antibiotic combinations (e.g., tobramycin and ciprofloxacin) with MMC against multidrug-resistant (MDR) isolates of *Acinetobacter baumannii* have been reported [4]. Domalaon et al. [5] demonstrated that MMC synergized with the antibiotic combination tobramycin–ciprofloxacin against MDR clinical isolates of *Pseudomonas aeruginosa*, *Acinetobacter baumannii*, *Escherichia coli*, *Klebsiella pneumoniae*, and *Enterobacter cloacae*, although it did not synergize with any of those antibiotics alone. Imipenem-resistant and persister strains of *K. pneumoniae* were eradicated in vivo in an infection model on *Galleria mellonella* by using the synergetic combination of MMC with the conventional antibiotic imipenem and the lytic phage vB_KpnM-VAC13 [6]. MMC in combination with pentamidine or gentamicin has demonstrated a superior antibacterial effect compared with monotherapies on MDR *P. aeruginosa* in vitro and in vivo [7]. Several other MMC-antibiotic combinatorial synergetic combinations have been described, including MMC with trimethoprim against uropathogenic *E. coli* K12 strain MG1655 [8]. However, the same authors show that this synergetic combination increases the bacterial mutation rate, calculated from the number of resistant colonies identified, by at least 3-fold. Furthermore, *Staphylococcus aureus* pre-exposed to MMC showed a 16-, 32-, and 42-fold increase in the MICs (minimum inhibitory concentrations) of ciprofloxacin, vancomycin, and levofloxacin, respectively, when compared to the treatment with the antibiotics alone being this phenomenon attributed to changes in the lipid concentration of the treated cellular membranes, overproduction of the extracellular matrix, and stimulated bacterial aggregation to promote surface colonization [9]. Other authors demonstrated that MMC alone promotes horizontal dissemination of antibiotic resistance genes [10]. In this regard, MMC has been reported to activate the SOS response in bacteria, promoting the selection of antibiotic-resistant mutants [11]. Therefore, the use of human-safe doses of MMC can synergize with antibiotics, lowering the doses required to exert their antimicrobial action, but it might also promote, counterproductively, a selection of bacteria-resistant strains. In addition, in several infections the use of antibiotics is not recommended, for instance in the management of topically infected open wounds. In those pathologies, topical antibiotics might induce allergic or sensitivity reactions, promote resistance, and disrupt the physiological healing process, and their use is restricted to those severe cases where the bone can be compromised (i.e., osteomyelitis) [12].

To potentially avoid the possibility of antibiotic resistance development, we propose the combinatorial use of MMC with the wide-spectrum antiseptic octenidine (OCT) because, compared to traditional antibiotics, it has been reported that the chances of developing resistance to this antiseptic are reduced due to its non-specific mode of action via membrane disruption [13]. It has been described that Gram-positive multidrug-resistant *Staphylococcus aureus* (MRSA) [14] and Gram-negative *P. aeruginosa* [13] bacteria can adapt to high concentrations of OCT, but this modification is not stable and is lost when OCT is not present. It has been reported that this adaptation is achieved by mutating key residues in the SmvRA system (a transcriptionally regulated, inner membrane-associated efflux system) for Gram-negative bacteria [15]. Lescat et al. [16] showed that on four Enterobacterales species (i.e., *K. pneumoniae*, *K. oxytoca*, *E. coli*, and *E. cloacae*), different mutants were identified when subjected to in vitro selective adaptation to chlorhexidine, but this effect was lacking when subjected to OCT. In addition, mutants with high MICs for chlorhexidine were identified for all four species, with cross-resistance detected for colistin (i.e., a polymyxin antibiotic), while OCT susceptibility remained unchanged. Several other advantages of OCT include its residual efficacy after treatment [17,18], rapid onset, broad-spectrum antimicrobial activity even in the presence of common interfering substances present in blood and in mucous membranes [19], and anti-inflammatory and protease-inhibitory ability when applied on treated wounds [20].

Pharmaceutical products are widely used compounds in healthcare settings which might carry the risk of release into the aquatic environment, especially through hospital effluents and wastewater, where they may persist due to their chemical stability and biological activity. Some cytotoxic drugs such as cytarabine, etoposide, gemcitabine, iphosphamide, and methotrexate have been quantified at concentrations above 464 ng/L [21] because current remediation technologies are not able to remove some of these compounds in their entirety [22]. The presence of these drugs raises concerns about possible adverse effects on aquatic organisms and on the disruption of key ecosystem functions [23]. Due to their increasing use and persistence in the environment, some drugs are classified as contaminants of emerging concern (CECs). This means that they are newly identified compounds in the environment, whose presence and potential impact are not yet systematically regulated or monitored, but pose an environmental and health risk.

To the best of our knowledge, there are no specific works on the environmental impact of MMC, although it is considered an emerging contaminant due to its physicochemical properties, which could be persistent in the environment and could present risks derived from its toxicity, bioaccumulation, and potential to affect aquatic organisms and the food chain [24]. On the other hand, the increasing use of OCT and its presence in aquatic environments can potentially lead to resistance development in bacteria such as *P. aeruginosa* [13] and modify efflux pumps [25], which raises uncertainties about its environmental toxicity and possible cumulative effects with critical implications for public health and microbial ecology [26]. Although no published data have reported the detection of MMC or OCT in river or surface waters at environmental concentrations, nor in global monitoring studies of pharmaceutical contaminants in the aquatic environment, this underscores the importance and originality of investigating their potential environmental impact and applying specific ecotoxicological assessment methods for their detection. Therefore, ecotoxicological assessment using standardized bioassays with *Daphnia magna* and *Aliivibrio fischeri* is essential as a starting point to characterize their potential impact on the environment.

Taking advantage of those previously reported benefits for OCT with the antimicrobial effect of MMC and their non-specific mechanisms of antimicrobial action, in this work we have demonstrated that a combination of OCT and MMC shows large antimicrobial efficacy against clinical strains of Gram-positive and Gram-negative pathogenic bacteria in their planktonic and sessile forms, and, at equivalent doses, subcytotoxic effects on human cell lines. We have also demonstrated the high efficacy of those combinations against intracellular bacterial persistence. Moreover, the ecotoxicological assessments conducted indicate that both compounds, particularly OCT, may pose environmental risks due to their acute toxicity in aquatic bioindicators. The response to the OCT-MMC combination depended on the bioindicator: lower toxicity was observed in *A. fischeri* compared with the individual compounds, whereas in *D. magna*, the mixture showed toxicity comparable to OCT alone. These findings highlight the importance of considering combined exposure scenarios and species-specific responses in environmental risk evaluations.

## 2. Results and Discussion

Table 1 shows MICs and MBCs for OCT and MMC against the two reference clinical strains MRSA USA300 and uropathogenic *E. coli*. The required doses to exert their antimicrobial action against both strains were lower for MMC compared to OCT. The superior efficacy of the former can be attributed to its selective antibiotic nature (being naturally produced by *Streptomyces caespitosus* and *Streptomyces lavendulae*) [27] whereas the antiseptic nature of OCT (i.e., a gemini cationic surfactant) lacking targeted specificity might be responsible for its lower antimicrobial ability. OCT, as mentioned before, binds to lipid components on the bacterial membranes, disrupting their structure and producing the leakage of intracellular components [9]. The large difference between the MIC and MBC values for OCT against MRSA observed might be attributed to a potential tolerance effect, but confirming this issue would require additional experiments, which are beyond the scope of the present manuscript. MMC was more effective against the pathogenic Gram-positive than against the Gram-negative one, whereas the opposite trend was observed for OCT, which represents a positive outcome to obtain wide-spectrum antimicrobial action when combined. This phenomenon can be attributed, on the one hand, to the presence of the outer membrane (i.e., lipopolysaccharide, LPS) in Gram-negative bacteria, which might hinder MMC diffusion. On the other hand, the different activity observed might be related to the different lipid composition of both bacteria. The saturated fatty acids, unsaturated fatty acids, and cyclopropane fatty acids differ for both bacteria [28]. In addition to the outermost LPS-based membrane, the inner membrane (plasma membrane) of *E. coli* contains phospholipids with a higher proportion of unsaturated fatty acids compared to those of *S. aureus*, and this can favor their interaction with OCT and the superior antimicrobial effect on *E. coli* observed. Our results are also in agreement with previous reports where MMC concentrations required to inhibit *S. aureus* ATCC 25923 growth (MIC: 3.25 µg/mL) were ~4 times lower than those required to inhibit *E. coli* BL21(DE3)pLys growth (MIC: 12.5 µg/mL). Also, our MIC values are close to those described by Denket et al. [29], who reported average MIC_90_ values of 2 and 4 µg/mL for OCT against bacterial isolates from different clinical samples of *S. aureus* (N = 24) and *E. coli* (N = 41).

As mentioned before, this different behavior in which one selected antimicrobial is more effective against Gram-positive bacteria and the other against Gram-negative bacteria is key to exert wide-spectrum antimicrobial action when they are used in combination. Many topical infections are polymicrobial, coexisting Gram-negative and Gram-positive bacteria in a symbiotic community. For instance, pulmonary infections and topical chronic wounds are frequently infected by *P. aeruginosa* and *S. aureus* simultaneously [30]. Virulence factors produced during co-infection make them more resistant to antimicrobial therapies and delay healing [31]. Several clinical examples describe the pathological challenge of polymicrobial topical infections where the causative pathogen includes *E. coli* and *S. aureus*, for instance diabetic foot ulcers, pressure ulcers, post-surgical infections, impetigo, etc. [32,33]. Herein, we took advantage of both antimicrobial effects; the alkylating effect of MMC was combined with the cationic and amphiphilic features of OCT, which disrupt microbial cell membranes, to eliminate both Gram-positive and Gram-negative bacteria.

**Figure 1:**
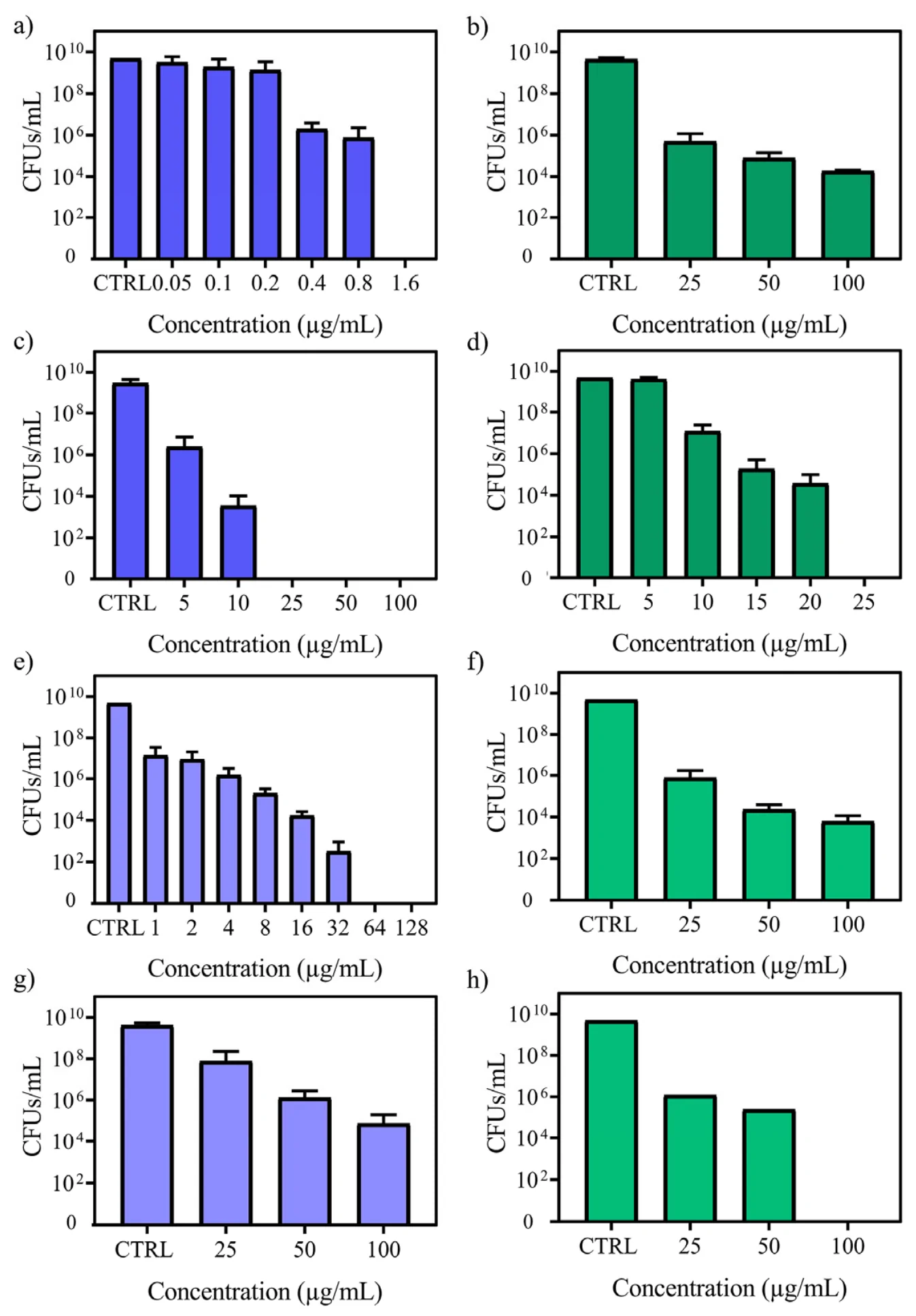
Antibiofilm activity of MMC and OCT against MRSA and *E. coli* UTI in the inhibition of biofilm formation assay (**a**–**d**) and in the eradication assay of pre-formed biofilms (**e**–**h**): (**a**) MBIC of MMC against MRSA, (**b**) MBIC of MMC against *E. coli* UTI, (**c**) MBIC of OCT against MRSA, (**d**) MBIC of OCT against *E. coli* UTI, (**e**) MBEC of MMC against MRSA, (**f**) MBEC of MMC against *E. coli* UTI, (**g**) MBEC of OCT against MRSA, and (**h**) MBEC of OCT against *E. coli* UTI. Results are expressed as mean ± SD from three independent experiments, each performed in duplicate.

The minimum biofilm eradication concentration (MBEC) and the minimum concentration to inhibit biofilm formation (MBIC) are depicted in Figure 1. We observed that MMC is able to inhibit MRSA biofilm formation at concentrations as low as 0.4 µg/mL and completely suppressed biofilm formation at 1.6 µg/mL (Figure 1a). On already formed mature biofilms, the concentration of MMC required to completely eliminate them was 64 µg/mL (Figure 1e). Compared to the MBC obtained for the bacteria in their planktonic form, the concentration required to inhibit biofilm formation or to eradicate mature biofilms of the same strain increased by 6.4- and 256-fold, respectively, which is indicative of the challenging scenario when sessile bacteria are formed. Against *E. coli* UTI, MMC was able to inhibit biofilm formation at 25 µg/mL and was unable to prevent its formation at the highest concentration tested (100 µg/mL) (Figure 1b). The same values were obtained on mature pre-formed *E. coli* UTI-derived biofilms (Figure 1f). Again, the doses required to inhibit biofilm formation or to produce an inhibitory effect on mature *E. coli* UTI biofilms were way larger than the MICs retrieved in its planktonic form, where only 0.75 µg/mL was enough to inhibit bacterial growth.

OCT against MRSA USA300 required a concentration of 25 µg/mL to completely prevent biofilm formation (Figure 1c), whereas on already formed mature biofilms, OCT was unable to eradicate them at the highest concentration tested (100 µg/mL) (Figure 1g). The same concentration (25 µg/mL) was therefore required to eradicate planktonic bacteria (MBC) and also to inhibit biofilm formation (MBIC). Once again, the detergent-like effect of the positively charged bispyridinamine OCT lacking specific bacterial targets could be responsible for this effect. OCT disrupts the bacterial cell membrane regardless of the form of the bacterium (i.e., biofilm-forming or planktonic) and efficiently permeates through the bacterial biofilm due to its reduced size. Several others have also shown that, at the same concentration, OCT is able to inactivate MRSA in its planktonic and sessile forms and even in the presence of serum proteins [34]. Against *E. coli* UTI, tested OCT was able to completely inhibit biofilm formation at 25 µg/mL (Figure 1d) and completely eradicate mature biofilms at 100 µg/mL (Figure 1h). The superior action of OCT against Gram-negative bacteria in its sessile form corroborated the results obtained in planktonic form, whose effect was attributed to the presence of the outer membrane (i.e., lipopolysaccharide, LPS) in Gram-negative bacteria and to the different lipid composition of both bacteria.

**Figure 2:**
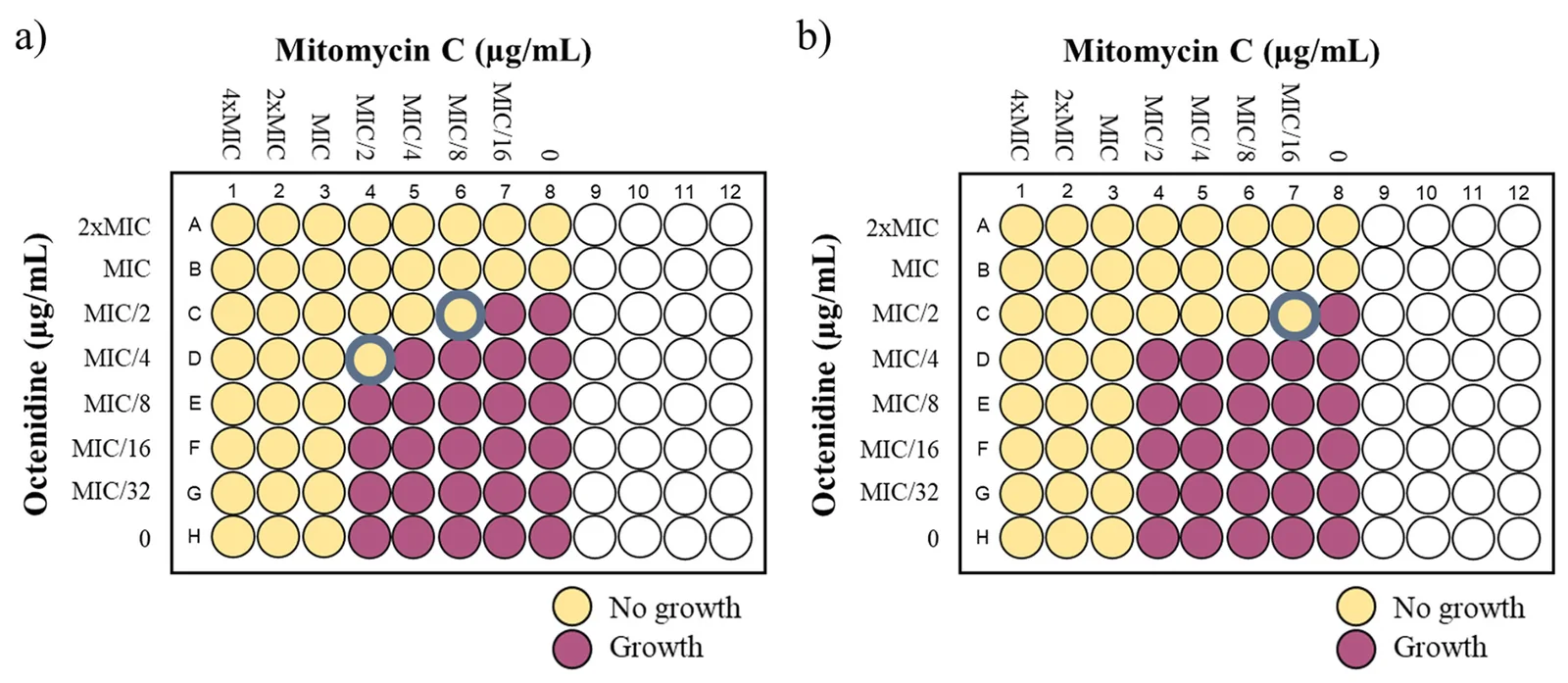
Schematic representation of the results obtained from the evaluation of the combinatory effect between MMC and OCT. Fractional inhibitory concentration index (FICI) analysis showing the additive effect of MMC and OCT: (**a**) against MRSA and (**b**) against *E. coli* UTI. All experiments were conducted in triplicate.

We therefore identified a combination of two antimicrobials that were able to exert bactericidal action against Gram-positive (MRSA) and Gram-negative (*E. coli* UTI) bacteria when free-floating and when forming biofilms. The checkerboard assay on the evaluation of the interaction between MMC and OCT revealed an additive effect with an FIC index between 0.5 and 2 for both bacteria (0.62 and 0.56 for MRSA and *E. coli*, respectively) (Figure 2). Although the interaction between MMC and OCT was classified as additive rather than synergistic, the combination still allowed the use of lower concentrations of both compounds while maintaining antibacterial activity. This may be particularly relevant as the use of lower concentrations of each compound could potentially reduce cytotoxicity while maintaining antimicrobial activity. The non-synergetic effect between common antiseptics has been widely demonstrated by other authors [35], attributed to the multiple mechanisms of antimicrobial action that differ from antibiotics, which usually have one specific target and are prone to synergetic interactions with other antimicrobials. Although species-specific, our results demonstrate, with the strains tested, that MMC and OCT show additive effects on those bacteria. As has been reported previously, OCT binds through hydrophobic and electrostatic interactions with the negatively charged bacterial membrane wall, causing permeabilization, neutralization, and disordering of the lipid bilayer and rendering cell lysis [36,37]. On the other hand, as reported in the literature, MMC alkylates DNA, inhibiting its synthesis. We hypothesize that this additive effect could potentially reduce resistance development, minimize toxicity, and finally improve treatment outcomes when polymicrobial infection might occur. Other additive and synergetic effects have been reported for OCT in combination with antibiotics such as disodium carbenicillin against *Neisseria gonorrhoeae* (ATCC 49226) [38], with natural products such as lavender essential oil against *S. aureus* ATCC 43300 (MRSA) [39]; complexing agents such as EDTA (ethylenediaminetetraacetic acid) against *P. aeruginosa* CIP (Collection de l’Institut Pasteur Paris) 103.467 and *S. aureus* CIP 4.83 [40]; and miconazole (an antifungal azole) against *S. aureus* SH1000 and the clinical isolate *S. epidermidis* CL7 [41].

**Figure 3:**
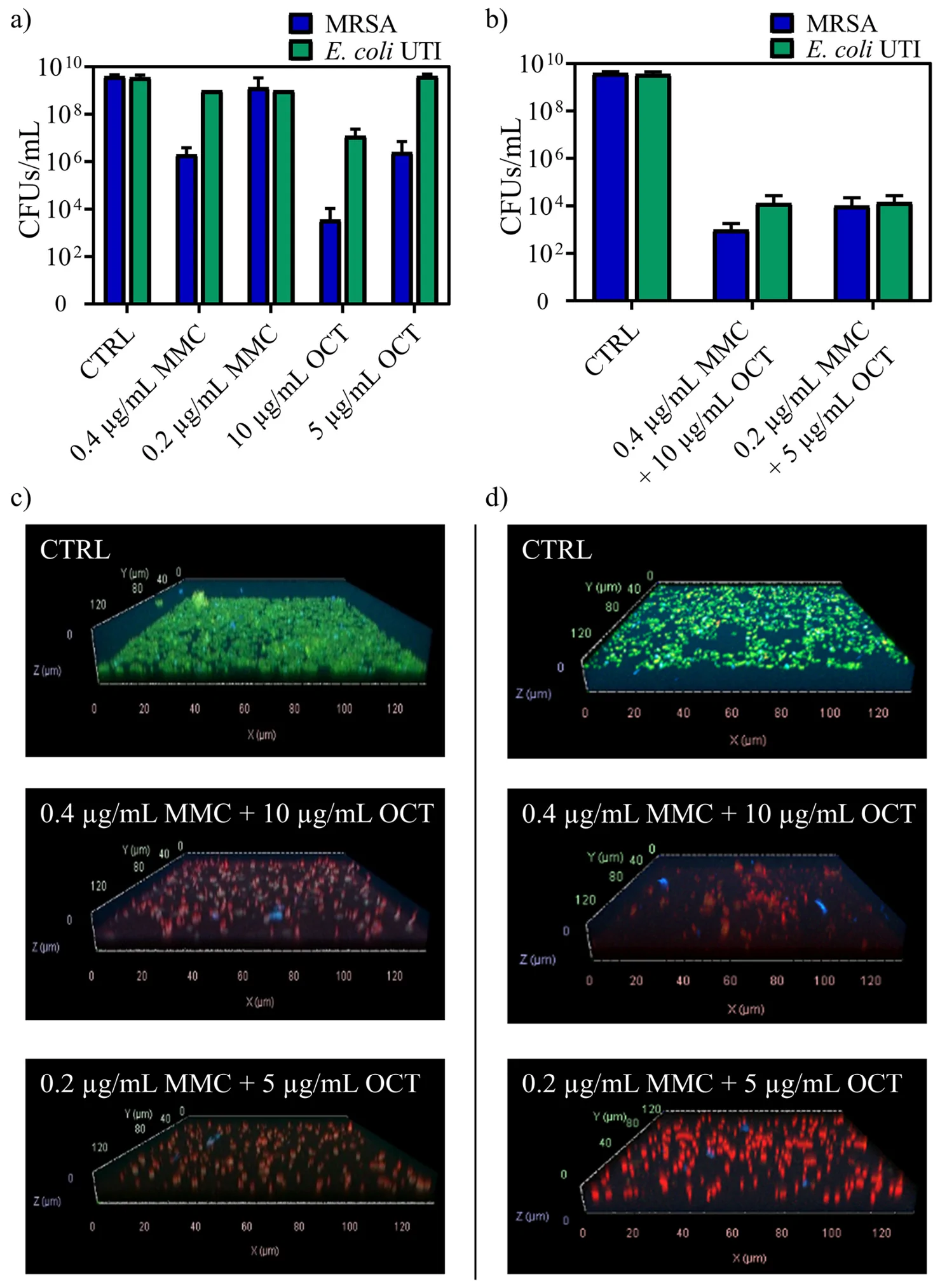
Inhibitory antibiofilm activity against MRSA (blue bars) and *E. coli* UTI (green bars): (**a**) after treatment with MMC and OCT individually at their MBICs, and (**b**) after combinatorial treatment with MMC and OCT, applied at minimum biofilm inhibitory concentrations (MBICs) and at additive concentrations. Confocal laser scanning microscopy images showing biofilm structure before and after combinatorial treatment with MMC and OCT, applied at minimum biofilm inhibitory concentrations (MBICs) and at additive concentrations of (**c**) MRSA and (**d**) *E. coli* UTI. Images show biofilm matrix stained with Calcofluor (blue), live bacteria (stained in green) and dead bacteria (stained in red). Results are expressed as mean ± SD from three independent experiments, each performed in duplicate.

Biofilm formation inhibition studies were conducted for both bacterial strains (MRSA and *E. coli* UTI) using the combination of the MBICs of both compounds against each strain. The selected concentrations were 0.4 µg/mL MMC + 10 µg/mL OCT, which represent the MBICs for MRSA and *E. coli* UTI, respectively. After confirming the additive effect on the antibacterial activity of both compounds, parallel studies were performed using half of the selected concentrations (0.2 µg/mL MMC + 5 µg/mL OCT), with the aim of reducing doses of the antibacterial agents as a potential strategy to combat the emergence of bacterial resistance and reduce cytotoxicity against eukaryotic cells. As shown in Figure 3a, except for OCT at its highest concentration (10 µg/mL), neither compound at the selected concentrations in monotherapy was able to inhibit biofilm formation in both strains simultaneously. However, in Figure 3b, the combination of both compounds was highly effective in inhibiting biofilm formation in both MRSA and *E. coli* UTI. The additive effect in biofilm inhibition was further confirmed, as reducing the selected concentrations of both compounds by half was equally effective, with no significant differences observed between treatments. Confocal laser scanning microscopy was performed to visualize the biofilm before and after each treatment to corroborate the obtained results. As shown in Figure 3c, MRSA perfectly formed biofilm in the absence of treatment, with all bacteria stained in green (viable bacteria) and the extracellular matrix (i.e., biofilm) stained in blue. Following both treatments, a significant reduction in the extracellular matrix and clear antibacterial effects were observed. Green bacterial signals were reduced, and damaged bacteria stained in red appeared. In the case of the *E. coli* UTI strain (Figure 3d), exactly the same effect was observed after both treatments. Therefore, it can be concluded that the combination of both compounds at the selected concentrations effectively inhibits biofilm formation in both bacterial strains. Furthermore, as observed in the planktonic study, halving the concentration of both compounds remained equally effective, confirming the additive effect of MMC with OCT and highlighting the potential applicability of this combination therapy for bacterial infections while minimizing the required doses of antibacterial compounds individually to achieve the same effect.

**Figure 4:**
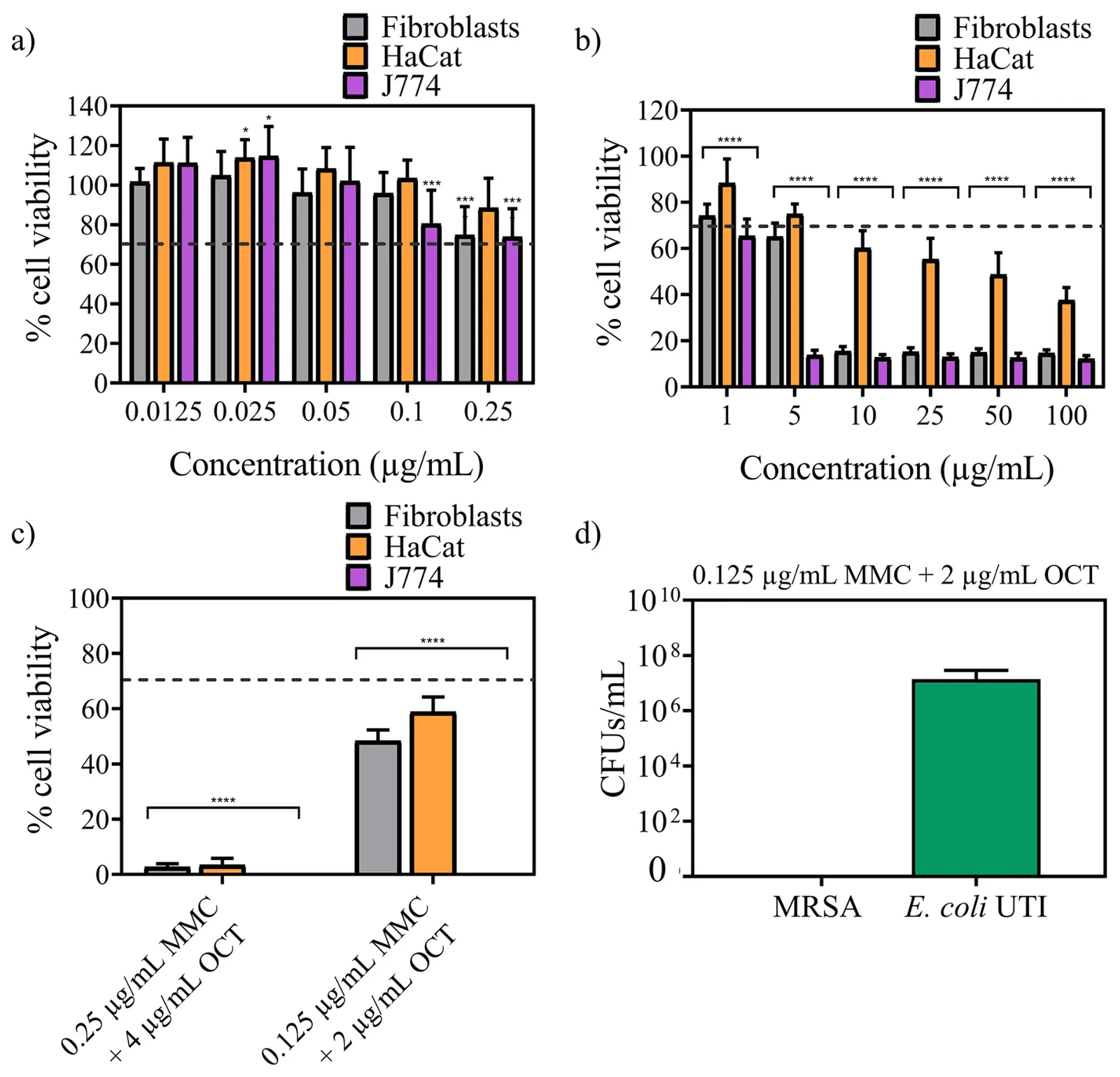
2D cytotoxicity assay in human dermal fibroblasts, HaCaT human keratinocytes, and J774A.1 mouse monocyte–macrophage cells of (**a**) MMC, (**b**) OCT, and (**c**) combined treatment for the eradication of planktonic bacteria and the additive treatment. Untreated cells were assigned 100% viability. Results are presented as mean ± standard deviation of at least three independent replicates. (**d**) Antibacterial activity of the combined treatment of MMC + OCT at the concentrations of 0.125 µg/mL MMC + 2 µg/mL OCT against MRSA and *E. coli* UTI. Results are expressed as mean ± SD from three independent experiments. The dashed line represents the 70% cell viability threshold defined by ISO 10993-5. Materials with a cell viability of 70% or higher are considered non-cytotoxic. Statistical differences between treated groups and the non-treated control (100% viability) are indicated by *p*-values: * *p* < 0.05; *** *p* < 0.001; **** *p* < 0.0001.

In the search for an antibiotic-like mixture which was able to eradicate bacteria but preserve eukaryotic cells (i.e., fibroblasts, keratinocytes and macrophages) viability, we evaluated their metabolic activity in traditional 2D cell cultures and also in fibroblast-based 3D spheroids for a potential topical application. We initially tested the MBC doses required to reach MBCs for MRSA and *E. coli* UTI, which were 0.25 and 4 µg/mL of MMC and OCT, respectively. According to the ISO 10993-5 standard applied to medical devices, a viability above 70% is considered the limit for considering a compound as non-cytotoxic. As it is shown in Figure 4a, in the 2D study, a dose of 0.25 µg/mL of MMC preserved viabilities over 70% for the three cell lines analyzed. Viabilities above 70% were reached for OCT on the three cell lines tested at doses below 5 µg/mL (Figure 4b). We then tested the bactericidal effect of this combinatory mixture of 0.25 µg/mL of MMC and 4 µg/mL of OCT on the pathogenic bacteria, and we observed that both bacteria were eliminated. However, the same mixture totally reduced the cellular viability. We halved the concentration of the mixture, and under those conditions, cellular viability remained above 50% (Figure 4c), whereas on bacteria we were able to eradicate MRSA but not *E. coli*, as shown in Figure 4d.

**Figure 5:**
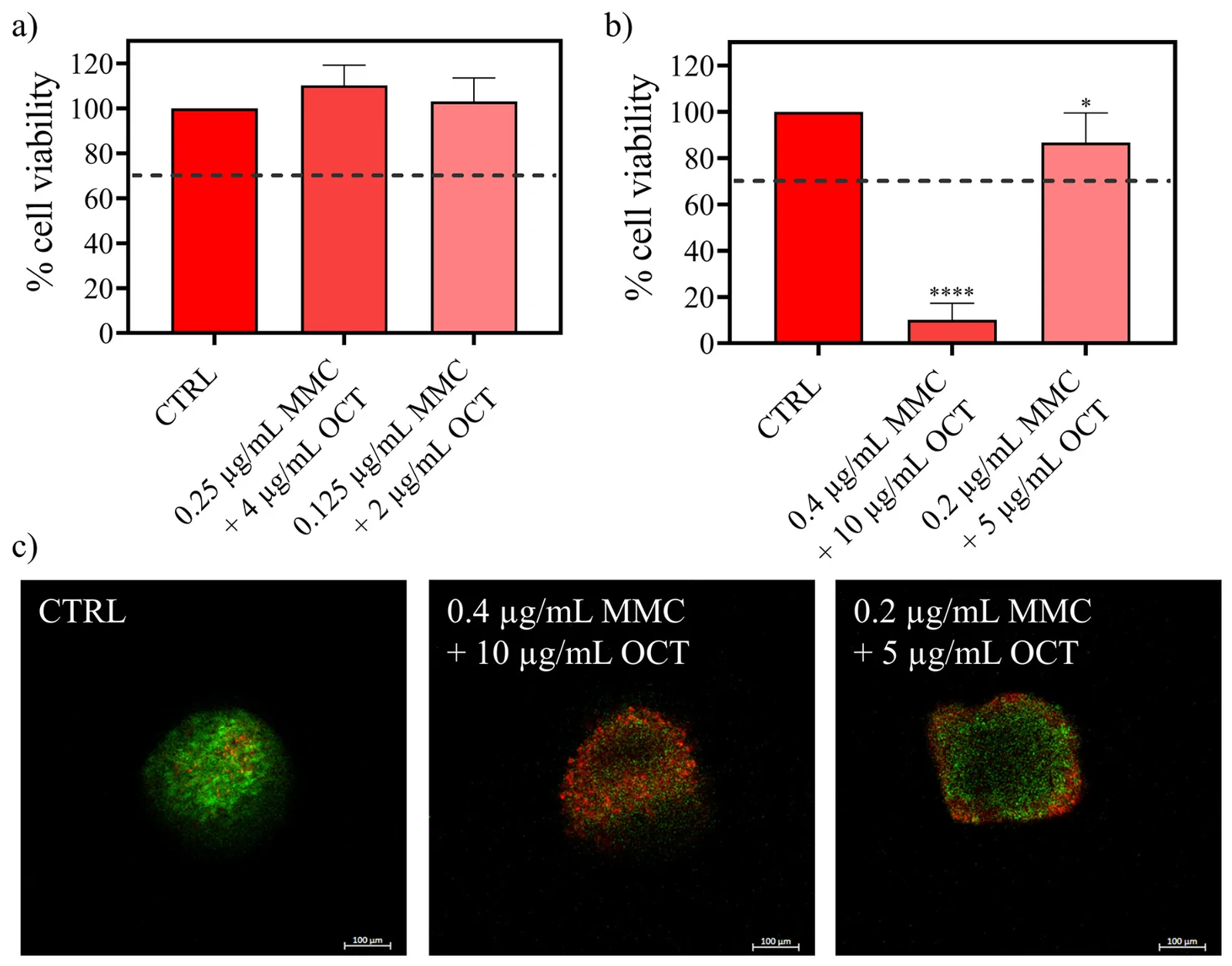
The 3D cytotoxicity in human dermal fibroblast spheroids: (**a**) cell viability of the combinatorial treatment at the MBCs determined in planktonic cultures and of the additive concentrations for the eradication of MRSA and *E. coli* UTI; (**b**) cell viability of the combinatorial treatment at the biofilm formation inhibitory concentrations for MRSA and *E. coli* UTI and of the additive concentrations; (**c**) confocal laser scanning microscopy images of untreated human dermal fibroblast 3D spheroids and after combinatorial treatment at biofilm formation inhibitory concentrations for MRSA and *E. coli* UTI, as well as at additive concentrations. Images show live cells (green) and dead cells (red). Results are expressed as mean ± SD from three independent experiments. The dashed line represents the 70% cell viability threshold defined by ISO 10993-5. Materials with a cell viability of 70% or higher are considered non-cytotoxic. Statistical differences between treated groups and the non-treated control (100% viability) are indicated by *p*-values: * *p* < 0.05, and **** *p* < 0.0001.

We then tested the cellular viability on 3D spheroids that were prepared using the hanging drop method. Compared to traditional 2D cell cultures, 3D cultures represent a more physiologically relevant microenvironment where the complexity of tissue architecture and the nutrients and oxygen diffusion gradients resemble the in vivo tissue heterogeneity. In this model, we observed, at the selected doses, no cytotoxic effects for both of the MMC/OCT mixtures tested by measuring the redox activity during the aerobic respiration of metabolically active cells (Figure 5). This reduced cytotoxicity in 3D compared to traditional 2D cell cultures has been widely reported on many different cell lines, including skin cells, and also corroborates that 3D spheroids represent more accurately real physiological responses to xenobiotic compounds [42]. This reduced cytotoxicity observed in the 3D spheroids could be explained by limited drug penetration through the extracellular matrix, diffusion limitations, which create a gradient across the spheroid, and the structural and metabolic heterogeneity caused by the extracellular matrix in the 3D spheroid. Overall, these differences may explain why the 3D fibroblast spheroid model showed lower cytotoxicity than the 2D cell culture model, suggesting that 3D models may be more suitable for cytotoxicity studies. The use of 3D spheroids over the traditional 2D cell cultures has been recommended by other authors when evaluating the topical effects of antiseptics [43]. The reduced cytotoxicity observed in 3D fibroblast cultures was also corroborated by confocal fluorescence microscopy, as shown in Figure 5c. By using the membrane-permeable live-cell labeling dye, calcein-AM, we were able to visualize alive cells in the 3D spheroid with similar fluorescence for the MMC-OCT-treated samples (at the lowest concentrations) as that retrieved for untreated controls. The signal retrieved from the ethidium homodimer-1, which labels dead cells as having damaged plasmatic membranes, again produced a similar fluorescent signal for the MMC-OCT-treated samples (at the lowest concentrations) and for the untreated controls. Although MMC is a cytotoxic anticancer drug, it has been used topically in clinical practice at low concentrations and for short exposure times [44,45]. For instance, MMC is widely used in ophthalmology as a topical adjunct to reduce postoperative scarring and improve surgical outcomes because topical application results in minimal systemic absorption [46]. However, its potential toxicity should be carefully considered. Further studies would be needed to guarantee the safe use of the selected combinations at specific doses and to guarantee their non-systemic absorption after topical administration. In our study, the additive effect of the MMC/OCT combination may allow the use of lower MMC concentrations while maintaining antibacterial activity, which could help reduce cytotoxicity. However, more studies are needed to evaluate its long-term safety, the most appropriate dose, and its potential clinical use in the management of topical infected wounds.

As we mentioned before, one of the huge advantages of MMC is its reported ability to eradicate intracellular bacterial pathogens independently of their bacterial metabolism, including pathogenic *E. coli* O157:H7 and MRSA [3]. MRSA spends part of its life cycle intracellularly as a means of survival through varied phenotypes (e.g., small colony variants, SCVs) [47]. When exiting the intracellular environment, the SCVs can quickly return to their fully virulent wild-type form, potentially triggering a new infection episode. Compared to untreated controls, 3D spheroids also showed large viability at all mixed concentrations except when the OCT concentration was increased to 10 µg/mL, where large stained regions in the spheroid would be an indication that the dye has permeated through disrupted plasma membranes, staining intracellular DNA (Figure 5c). This would indicate that against planktonic bacteria, 4 µg/mL OCT and 0.25 µg/mL MMC would exert a large antimicrobial action against the two model bacteria while preserving 3D fibroblast-derived spheroids’ viability. On the inhibition of biofilm formation, the different mixtures tested were unable to hinder biofilm formation; however, they were more effective at inhibiting the development in both strains than each compound by itself (Figure 3a,b). On the other hand, planktonic bacteria are responsible for the first stage of infection, while they may eventually form biofilms; eradicating them early as prophylaxis can prevent the development of more serious infections or complications. Biofilm formation occurs over time; therefore, by addressing planktonic bacteria, we would reduce the number of bacteria that have the potential to form biofilms. We would also reduce the chances for bacteria to spread to new areas and reduce the overall impact of the potential infection.

**Figure 6:**
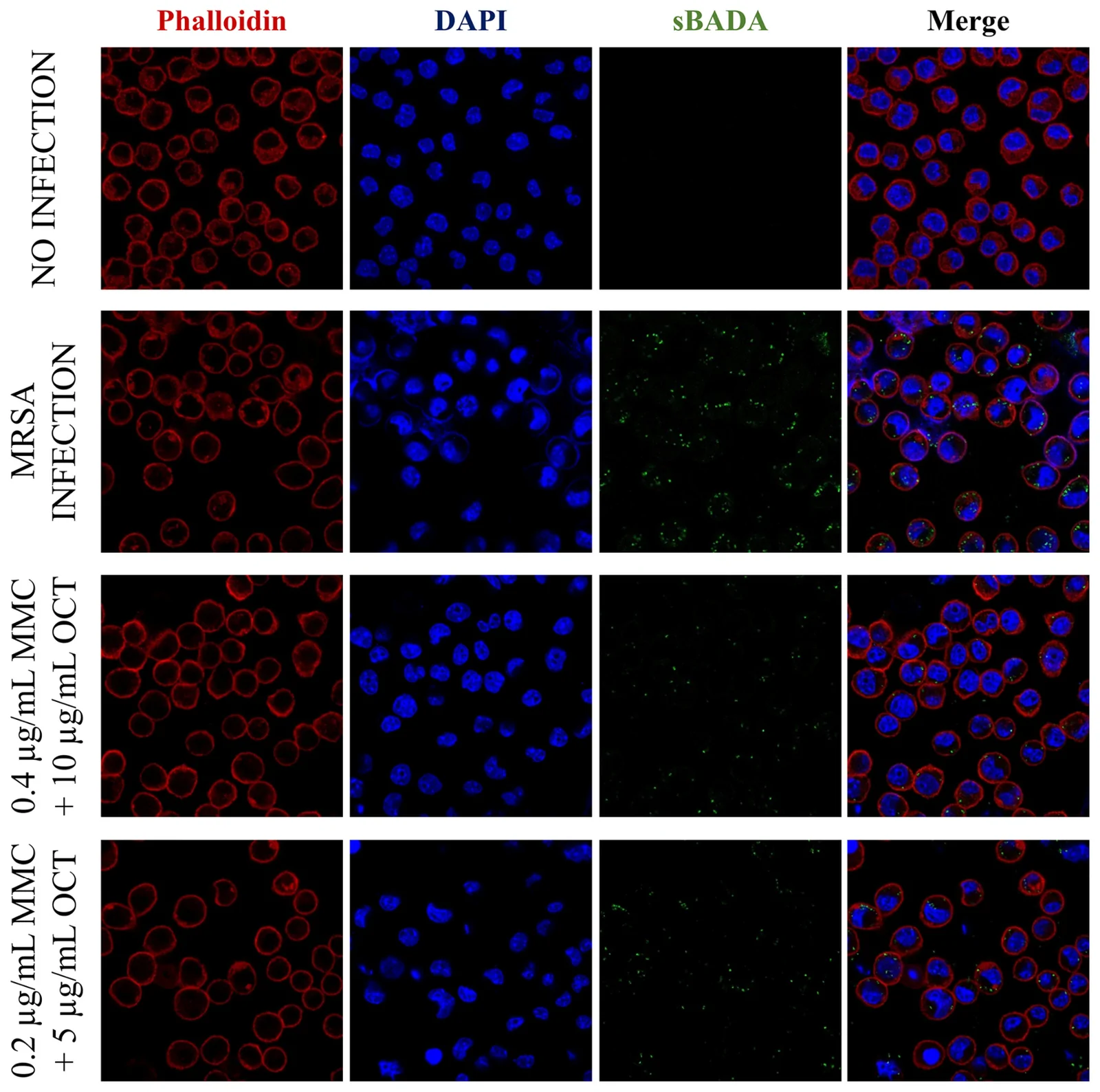
In vitro co-culture model of MRSA and J774 macrophages. Images correspond to uninfected J774 cells (first row), infected cells without treatment (second row), and cells treated for 4 h with the combinatorial biofilm-inhibitory concentrations of MMC + OCT (third row), as well as with the additive concentrations (half of the concentrations) of MMC + OCT (fourth row). Bacteria are shown in green (sBADA), the cytoskeleton of J774 appears in red (phalloidin 546), and cell nuclei appear in blue (DAPI), using a 63× objective.

As shown in Figure 6, J774 macrophages were successfully infected with MRSA, as evidenced by the presence of bacteria with membranes stained by the fluorescent amino acid sBADA (green dots) within the macrophages’ cytoplasm, confirming intracellular infection. In the images of uninfected cells, no bacterial presence was observed inside macrophages, whereas in the infected cell images, bacteria were detected intracellularly in nearly all macrophages, which preserved their typical morphology. Treatment with the biofilm-inhibitory concentrations of 0.4 µg/mL MMC + 10 µg/mL OCT resulted in a marked reduction in intracellular bacterial burden, and this reduction in bacterial viability was equally efficient when the tested concentrations were halved, reflecting the additive effect of the two compounds. As previously demonstrated in the in vitro assays shown in Figure 4b, the tested concentration of the antiseptic OCT was cytotoxic for this cell line. For this reason, incubation with both compounds could not be extended to 24 h to further assess their efficacy. Nevertheless, after only 4 h of treatment, bacterial load was significantly reduced, confirming the effectiveness of the selected compounds in an in vitro infection model at concentrations required to inhibit biofilm formation of both MRSA and *E. coli* UTI strains simultaneously. Importantly, the additive effect of the two compounds should be highlighted, since half of the selected concentrations proved equally effective against intracellular infection, reducing cellular cytotoxicity, as demonstrated in the 3D model.

**Figure 7:**
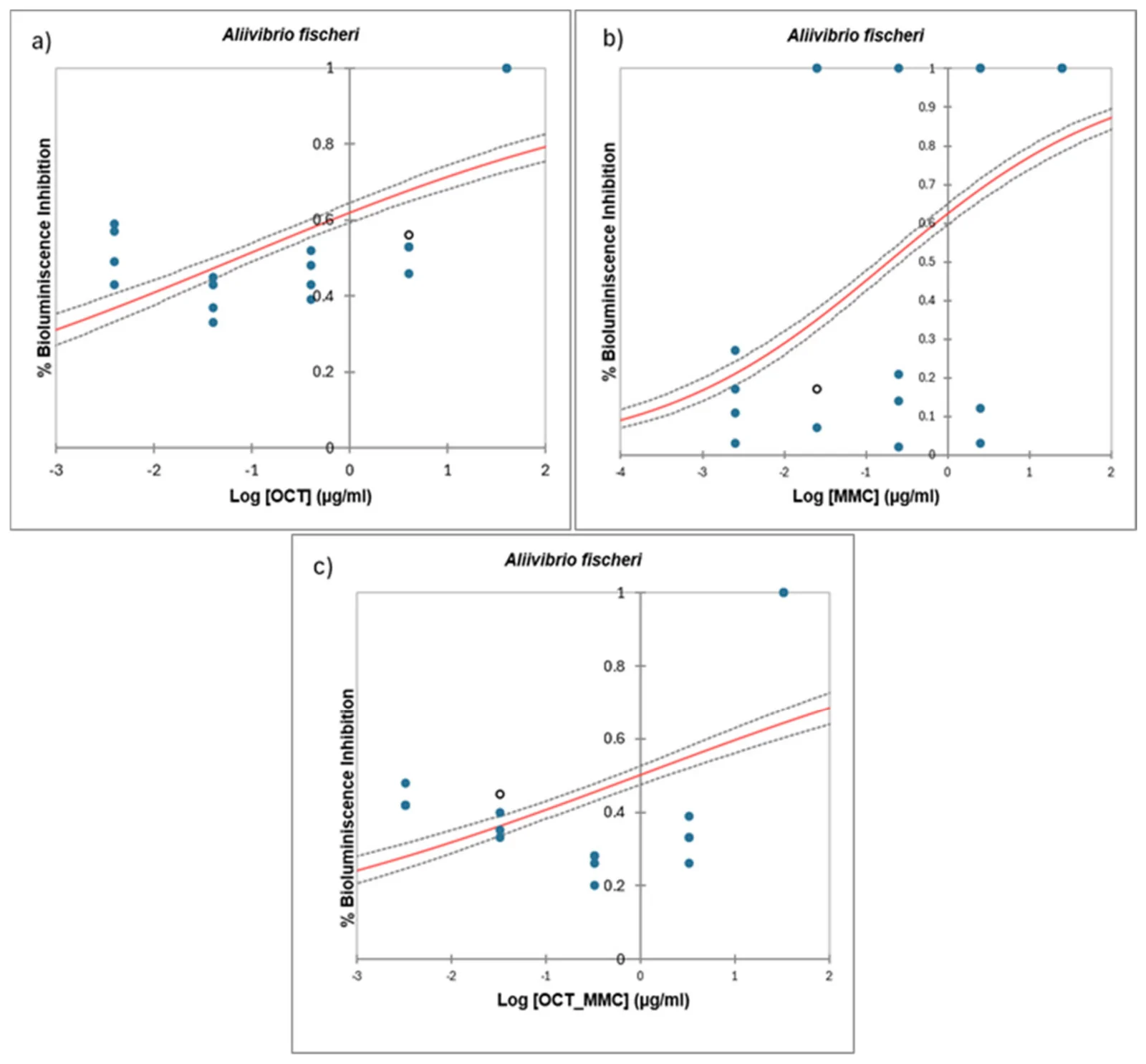
Dose–response curve for *Aliivibrio fischeri* after 30 min exposure to: (**a**) OCT, (**b**) MMC, and (**c**) their combinations (OCT-MMC). The solid line represents the model; the dashed lines are the superior and inferior confidence limits (95%); and the dots represent the experimental data.

Figure 7 shows the dose–response curve for *A. fischeri* bioluminescence inhibition when these bacteria were exposed to OCT, MMC, and their combinations. Their EC_50_ values are shown in Table 2. According to the data obtained from these experiments, the combination of OCT and MMC was less toxic (EC_50_ = 2.082 µg/mL, CI = 1.155–3.995 µg/mL) than OCT and MMC when tested alone, while OCT was more toxic than MMC (Table 2). None of the dose–response curves exhibited a markedly steep slope, which would indicate a wide margin between sublethal and inhibitory concentrations. In addition, the shape of the curves was quite similar for the three experiments.

**Figure 8:**
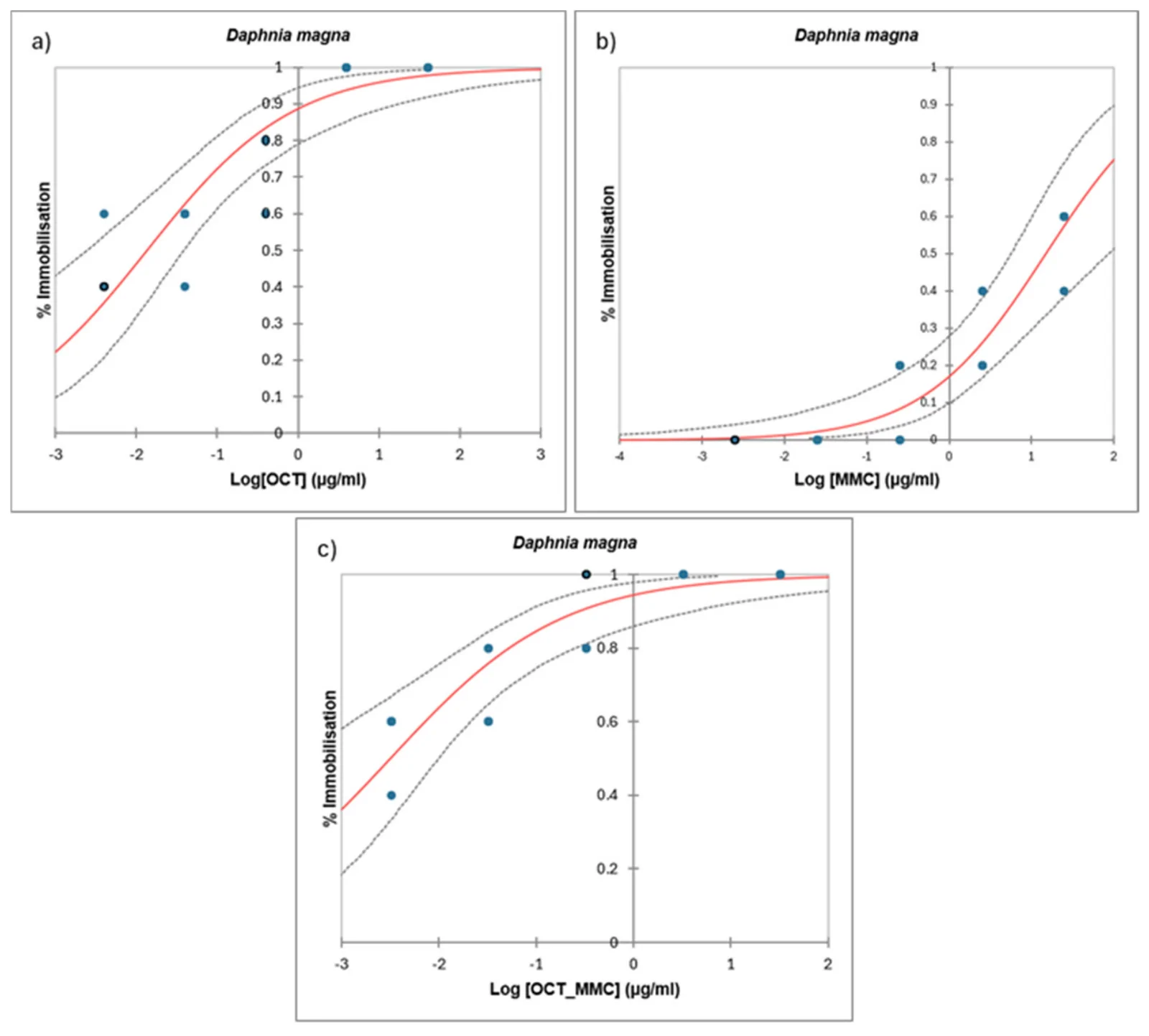
Dose–response curve for *Daphnia magna* after 24 h exposure to (**a**) OCT, (**b**) MMC, and (**c**) their combinations (OCT-MMC). The solid line represents the model; the dashed lines are the superior and inferior confidence limits (95%); and the dots represent the experimental data.

The second set of ecotoxicity bioassays focused on the effects of OCT and MMC exposure on *D. magna*, a standard model organism for evaluating freshwater invertebrate sensitivity to contaminants. Figure 8 shows the dose–response curves for the effect of OCT, MMC and their combinations on *D. magna*. In addition, specific EC_50_ values and confidence intervals are given in Table 2. Among the compounds tested, MMC showed a comparatively lower acute effect on *D. magna*. However, both OCT and its combination with MMC displayed lower EC_50_ values, and so, higher toxicity, which, at the same time, equal each other (EC_50_ close to 0.01 µg/mL, see Table 2). In fact, the shape of the dose–response curves for these two experiments (Figure 8a,c) was very similar, but they were different from the shape of the dose–response curve for MMC (Figure 8b).

As far as we know, no studies have been reported specifically to evaluate the ecotoxicological effects of MMC, OCT, or their combination on *D. magna* and *A. fischeri* or on other aquatic bioindicators. This highlights the novelty and originality of the present work.

MMC and OCT have different known modes of action, which may help contextualize the response observed in *A. fischeri*. MMC has been described as an alkylating agent that can interact with DNA, whereas OCT is known to act mainly through interactions with microbial membranes. Based on these established modes of action, differences in uptake, intracellular targets, or exposure kinetics could contribute to the bioindicator-dependent response observed in this study. However, DNA damage, membrane disruption, uptake kinetics, and specific metabolic effects were not directly measured in *A. fischeri* in the present work. Therefore, these mechanisms should be considered as background information and possible explanations rather than experimentally demonstrated processes in this assay [48,49].

The OCT-MMC mixture showed a higher EC50 in *A. fischeri* than the individual compounds, indicating lower toxicity of the mixture under the tested conditions. However, the present study was not designed to identify the mechanisms underlying this response. Therefore, possible explanations, such as changes in compound bioavailability, altered uptake, cellular stress responses, or non-additive interactions between both compounds, should be regarded as hypotheses rather than experimentally confirmed mechanisms.

The different acute toxicity profiles observed for MMC and OCT in *D. magna* may be related to their distinct known modes of action and exposure dynamics. OCT showed high acute toxicity in *D. magna*, which is consistent with its membrane-active properties described for other biological systems. However, specific membrane damage, uptake routes, and physiological alterations were not directly evaluated in this assay.

In contrast, MMC showed a lower acute effect on *D. magna* than OCT under the tested conditions. Nevertheless, the EC_50_ estimated for MMC should be interpreted cautiously, because its confidence interval extended beyond the highest concentration tested. Thus, rather than providing a precise toxicity threshold, this result indicates that MMC was less acutely toxic than OCT within the experimental range evaluated. Since genotoxicity, antioxidant responses, DNA repair, and compound accumulation were not assessed, any explanation involving these processes should be considered speculative and would require dedicated mechanistic or chronic exposure studies [50].

The OCT-MMC mixture exhibited an EC_50_ of 0.010 µg/mL, which was comparable to that of OCT alone (0.017 µg/mL), suggesting that OCT was the main driver of the acute toxicity observed in *D. magna* under the tested conditions. Previous studies on pharmaceutical mixtures in *D. magna* have reported deviations from simple additive toxicity, depending on the compounds tested, their modes of action, and exposure conditions [51]. Nevertheless, the present study does not allow us to determine whether physicochemical interactions, changes in bioavailability, altered uptake, pharmacodynamic interactions, or cellular defense responses contributed to the observed mixture response. Previous studies on pharmaceutical mixtures in *D. magna* have reported deviations from simple additive toxicity depending on the compounds tested, their modes of action, and exposure conditions [52,53]. Therefore, these mechanisms should only be considered as possible explanations rather than experimentally confirmed mechanisms.

The environmental risk posed by OCT and MMC, both individually and in combination, for aquatic organisms like *D. magna* and *A. fischeri* is significant and shaped by both the compounds’ distinct toxicities and their interactions. Ecotoxicity data show that OCT exerts very high acute toxicity in both bioindicators, especially on *D. magna* (CE_50_ = 0.017 µg/mL) and notably also in *A. fischeri* (CE_50_ = 0.063 µg/mL), indicating a relevant risk at environmentally realistic concentrations, capable of affecting vital functions and aquatic community structure according to ECHA (European Chemicals Agency) guidelines. In contrast, MMC shows low acute toxicity in *D. magna* (CE_50_ = 14.941 µg/mL) and moderate toxicity in *A. fischeri* (CE_50_ = 0.303 µg/mL), suggesting that the environmental risks of this compound are mostly relevant for chronic exposure scenarios, where its genotoxic action may impact reproduction and microbial survival. It is essential to keep in mind that sublethal effects, such as changes in reproduction, behavior, gene expression, and microbial community composition, can arise during chronic exposures and may not be fully captured by acute CE_50_ tests.

The combinations yield different outcomes: for *A. fischeri*, the combination reduces the toxicity of the separate compounds, though toxicity remains moderate; for *D. magna*, the acute toxicity of the mixture was comparable to that of OCT alone. This species-specific divergence underscores that environmental risk assessments for pharmaceutical mixtures must account for mechanisms of action, exposure routes, and bioindicator responses, as the simultaneous presence of antiseptics and cytotoxic compounds in water may not lead to additive effects, but rather risks modulated by antagonism dynamics and differing species sensitivity.

## 3. Materials and Methods

### 3.1. Materials

Mitomycin C (MMC) was purchased from GoldBio (St Louis, MO, USA). Paraformaldehyde (PFA) 4% in PBS, octenidine dihydrochloride 98% (OCT), and the LIVE/DEAD^TM^ Viability/Cytotoxicity Kit and the LIVE/DEAD™ BacLight™ Bacterial Viability Kit were obtained from Thermo Fisher Scientific (Waltham, MA, USA). Calcofluor White Stain and gentamicin sulphate were purchased from Sigma-Aldrich (Darmstadt, Germany). Dulbecco’s modified Eagle’s medium (DMEM) high glucose with stable glutamine, antibiotic-antimycotic solution (penicillin–streptomycin–amphotericin B; PSA) and Dulbecco’s Phosphate-Buffered Saline (DPBS) were acquired from Biowest (Nuaillé, France). Fetal bovine serum (FBS) was obtained from Gibco (Waltham, MA, USA), and the Blue Cell Viability reagent was purchased from Abnova (Taipei, Taiwan). Tryptic Soy Broth (TSB) and agarose were sourced from Condalab (Madrid, Spain).

Both the uropathogenic *E. coli* strain (*E. coli* UTI) and the clinical methicillin-resistant *S. aureus* USA300 (MRSA) strain were kindly provided by Dr. Cristina Prat (Institut d’Investigació en Ciènces de la Salut Germans Trias i Pujol, Badalona, Spain). The J774A.1 mouse monocyte–macrophage cell line (ATCC-TIB-67^TM^) was purchased from LGC Standards (Barcelona, Spain). HaCaT human keratinocytes were kindly donated by Dr. Pilar Martín-Duque (University of Zaragoza, Spain), and human dermal fibroblasts (NHDF-Ad) were sourced from Lonza (Basel, Switzerland).

### 3.2. Antimicrobial Activity of MMC and OCT

The antimicrobial activity of MMC and OCT was assessed against MRSA, as a model of a clinical Gram-positive bacterium and against *E. coli* UTI, as a model of a Gram-negative bacterium. Both strains were inoculated in TSB and cultured for 24 h at 37 °C under continuous shaking (150 rpm) until reaching the stationary growth phase (10^9^ CFU/mL). Cultures were then diluted in fresh TSB to achieve a concentration of 10^5^ CFU/mL and treated with MMC (0.025–0.25 µg/mL for MRSA and 0.5–3 µg/mL for *E. coli* UTI) or OCT (1–50 µg/mL for MRSA and 1–5 µg/mL for *E. coli* UTI) in TSB. Following 24 h incubation at 37 °C under continuous stirring (150 rpm), the antimicrobial activity was determined using the standard serial dilution method. The samples were plated on tryptone soya agar (TSA) plates to quantify viable bacteria via colony-forming units (CFU/mL) after 24 h of incubation. Both MIC (minimal inhibitory concentration) and MBC (minimal bactericidal concentration) were determined. Each experiment was performed three times in duplicate, with untreated bacterial samples serving as controls.

### 3.3. Synergy Studies by Fractional Inhibitory Concentration (FIC) Index Determination

The interaction between MMC and OCT was evaluated using the checkerboard microdilution method to determine the fractional inhibitory concentration (FIC) index. The experiments were performed in 96-well plates for each bacterial strain (MRSA and *E. coli* UTI). A stock solution containing four times the MIC for both compounds was prepared. OCT was serially diluted two-fold along the vertical axis, while MMC was serially diluted two-fold along the horizontal axis. Bacterial cultures were diluted to 10^6^ CFU/mL, and a volume of 20 µL was added to each well. Afterwards, the plates were incubated for 24 h at 37 °C with continuous shaking (150 rpm).

Two approaches were followed to evaluate bacterial viability. Firstly, sample turbidimetry was measured at 600 nm using a Varioskan LUX microplate reader (Thermo Scientific, Waltham, MA, USA). Secondly, sample fluorescence was assessed using the Blue Cell viability assay. For this, the plates were centrifuged for 5 min at 200 rcf. Then, the medium was discarded, and the reagent (10% (*v*/*v*) in fresh TSB) was added to each well. After 10 min of incubation at room temperature, fluorescence was measured using a Varioskan LUX microplate reader (535/590 nm ex/em). All experiments were performed in triplicate, and the results were compared to control samples (fresh TSB without bacteria for turbidity evaluation and cell viability assay reagent in fresh TSB for fluorescence assessment).

FIC index values were calculated as follows:

$$FIC\;index={FIC}_{OCT}+{FIC}_{MMC}$$

where

$${FIC}_{OCT}=\frac{{MIC}_{OCT}\;in\;presence\;of\;MMC}{{MIC}_{OCT}}$$

$${FIC}_{MMC}=\frac{{MIC}_{MMC}\;in\;presence\;of\;OCT}{{MIC}_{MMC}}$$

The interaction was classified as synergy (FIC index < 0.5), additive (0.5 ≤ FIC index ≤ 2), indifference (2 < FIC index ≤ 4) or antagonistic (FIC index > 4).

### 3.4. Biofilm Inhibition and Disruption Assays

The effects of MMC and OCT on MRSA and *E. coli* UTI biofilms were evaluated in terms of both biofilm formation inhibition and preformed biofilm disruption. Bacterial strains were inoculated in TSB and incubated for 24 h at 37 °C under 150 rpm of stirring rate to reach the stationary growth phase. The cultures were then diluted in fresh TSB to achieve a concentration of 10^7^ CFU/mL and then transferred to 96-well plates.

For the assessment of biofilm formation inhibition, the diluted cultures were treated with MMC (0.05–1.6 µg/mL for MRSA and 25–100 µg/mL for *E. coli* UTI) or OCT (5–100 µg/mL for MRSA and 5–25 µg/mL for *E. coli* UTI) in TSB, followed by incubation at 37 °C for 24 h without shaking to allow biofilm formation.

For the assessment of biofilm disruption, preformed mature biofilms were first established by incubating the diluted cultures mentioned before under identical conditions for 24 h. After washing with sterile PBS to remove planktonic cells, biofilms were treated with MMC (1–128 µg/mL for MRSA and 25–100 µg/mL for *E. coli* UTI) or OCT (25–100 µg/mL for MRSA and 25–100 µg/mL for *E. coli* UTI) for an additional 24 h at 37 °C.

In both experiments, biofilms were subsequently washed with sterile PBS, disrupted by sonication in an ultrasonic water bath, and serially diluted for CFU quantification after 24 h incubation. Experiments were conducted three times in duplicate, with untreated bacteria serving as controls.

The combined effects of both compounds on biofilm inhibition were also evaluated using four different combinations of OCT and MMC: (1) 4 µg/mL OCT + 0.25 µg/mL MMC, (2) 2 µg/mL OCT + 0.125 µg/mL MMC, (3) 10 µg/mL OCT + 0.4 µg/mL MMC, and (4) 5 µg/mL OCT + 0.2 µg/mL MMC. Bacterial colony counting was performed as previously described, and biofilm morphology was analyzed using confocal microscopy (Confocal Zeiss LSM 880 with Airyscan, Carl Zeiss, Germany). To do so, the biofilm extracellular matrix was treated for 1 min with Calcofluor White stain, and the bacteria were labeled with the LIVE/DEAD™ BacLight™ Bacterial Viability Kit (Thermo Fisher Scientific, Eugene, OR, USA) following the manufacturer’s instructions. Then, biofilms were fixed with 4% PFA in PBS. After 45 min of incubation, samples were mounted for further visualization by confocal microscopy with a 63× objective.

### 3.5. Cell Cytotoxicity Assays

The cytotoxic effects of MMC, OCT, and their combinations were evaluated in human dermal fibroblasts (NHDF-Ad), human keratinocytes (HaCaT), and mouse monocyte–macrophage cells (J774). Cells were cultured in DMEM High Glucose with stable glutamine, supplemented with 10% (*v*/*v*) FBS and 1% (*v*/*v*) PSA, at 37 °C and 5% CO_2_. Cells were seeded in 96-well microplates (18,000 cells/cm^2^) and incubated for 24 h before exposure to varying concentrations of MMC (0.25–2 µg/mL), OCT (1–200 µg/mL), and two different combinations of both: (1) 4 µg/mL OCT + 0.25 µg/mL MMC and (2) 2 µg/mL OCT + 0.125 µg/mL MMC. After a 24 h incubation, viability was assessed using the Blue Cell viability assay following the manufacturer’s instructions. Briefly, cells were incubated for 4 h with 100 µL of fresh supplemented DMEM containing 10% (*v*/*v*) of the reagent. After this, the fluorescence was measured at 570/590 nm (ex/em) using a Varioskan LUX microplate reader. The experiments were conducted in triplicate, with untreated cells serving as controls (100% viability).

### 3.6. Fibroblast Spheroid Formation and Evaluation of Additive Effects

The cytotoxic effects of the combined MMC and OCT treatment were also assessed in human dermal fibroblast (NHDP-Ad) spheroids, generated using the hanging-drop method. To form spheroids, 25 µL drops of supplemented DMEM High Glucose, each containing 10,000 cells, were placed on the lid of a Petri dish, with the bottom filled with sterile water or PBS to prevent evaporation. After 72 h, spheroids were collected, transferred to a 96-well microplate pre-coated with 50 µL of agarose (1% (*w*/*v*)), and incubated for 24 h at 37 °C and 5% CO_2_. The following combinations of OCT and MMC were then added: (1) 4 µg/mL OCT + 0.25 µg/mL MMC, (2) 2 µg/mL OCT + 0.125 µg/mL MMC, (3) 10 µg/mL OCT + 0.4 µg/mL MMC, and (4) 5 µg/mL OCT + 0.2 µg/mL MMC. After a 24 h incubation, spheroids were collected and washed with sterile PBS. To evaluate spheroid viability, two independent assays were performed. Firstly, cell viability was assessed using the Blue Cell viability assay. The medium was replaced with 100 µL of fresh supplemented DMEM containing 10% (*v*/*v*) of the reagent, and after a 24 h incubation (37 °C, 5% CO_2_), fluorescence was measured at 570/590 nm (ex/emission) using a Varioskan LUX microplate reader. Secondly, cell viability was analyzed using the LIVE/DEAD^TM^ Viability/Cytotoxicity Kit for mammalian cells following the manufacturer’s instructions. Briefly, a solution containing 5 µL of 4 mM calcein AM and 20 µL of 2 mM ethidium homodimer-1 (EthD) was added to the spheroids, followed by a 45 min incubation. After fixation with 4% PFA in PBS for 15 min, samples were analyzed using confocal microscopy with a 10× objective.

### 3.7. In Vitro Model of Intracellular Bacterial Infection and Treatment Evaluation

To assess the efficacy of the combined MMC and OCT treatment against MRSA, an in vitro infection model was established using J774 macrophages. Firstly, MRSA was inoculated in 4 mL of TSB and incubated at 37 °C with shaking for 24 h. When the stationary phase was reached, bacteria were diluted to mid-log phase (DO_600_ ≈ 0.6) and incubated with 1 mM of the fluorescent label sBADA (BODIPY-FL-conjugated D-alanine obtained from Bio-Techne R&D Systems, Minneapolis, MN, USA) for 24 h at 37 °C with agitation to ensure complete labeling of the bacterial cell wall for subsequent tracking. Then, cells were seeded in μ-dish 35 mm ibiTreat plates (130,000 cells/cm^2^) and incubated for 24 h in supplemented DMEM High Glucose. Prior to infection, bacteria were washed twice with PBS by centrifugation at 5000 rpm × 5 min to remove the non-incorporated dye. Labeled bacteria were dispersed in PBS to achieve a concentration of 10^7^ CFU/mL. Then, macrophages were washed with PBS, and supplemented DMEM was replaced with PSA-free medium to prevent any interference with the infection. Macrophages were then infected with MRSA at a multiplicity of infection (MOI) of 10:1, followed by a 30 min incubation at 37 °C. The medium was then discarded, and cells were washed with PBS to remove non-phagocytized bacteria. Infected cells were treated with gentamicin sulphate (100 µg/mL) for 1 h to eliminate any extracellular bacteria. Subsequently, the following treatments were applied to the co-cultures for 3 h, all prepared in PSA-free medium: (1) 4 µg/mL OCT + 0.25 µg/mL MMC and (2) 2 µg/mL OCT + 0.125 µg/mL MMC.

To determine treatment efficacy against intracellular bacteria, confocal microscopy was used. For this, the bacterial membrane was first labeled with a fluorescently tagged amino acid (sBADA) for 24 h. Then, cells were infected, treated, and fixed with 4% PFA for 45 min at room temperature and washed with PBS. After fixation, cells were washed with 1% PBS-BSA and with 0.1% saponin in PBS-BSA solution. Then, samples were incubated with Phalloidin 546 (1:200 in PBS-BSA-saponin solution) at room temperature for 1 h in dark conditions. Cells were washed with PBS-BSA solution and distilled water. Finally, samples were incubated with DAPI for 30 min at room temperature and were mounted in Mowiol mounting medium (Thermo Fisher Scientific, USA), and samples were analyzed using confocal microscopy.

### 3.8. Statistical Analyses

All data reported are expressed as mean ± standard deviation (SD). Statistical analyses of experimental data were conducted using Prism 8 software (GraphPad Software Inc., San Diego, CA, USA). Each experiment was performed in three independent replicates. For cellular experiments, statistical significance was determined using a two-way analysis of variance (ANOVA) followed by Dunnett’s multiple comparisons test to compare each experimental group with the control group, with differences considered significant at *p* ≤ 0.05.

### 3.9. Ecotoxicity Evaluation

#### 3.9.1. *Daphnia magna* Acute Immobilization Test

One of the bioindicators used to assess the environmental impact of MMC, OCT and their combinations in water was the freshwater crustacean *Daphnia magna*. The Daphtoxkit FTM distributed by Vidrafoc (Barcelona, Spain) was used to carry out this test. The kit contains the ephippia of *D. magna*, the culture medium in which the *Daphnia* develops and where the test is performed (synthetic freshwater (ISO 6341, 2012) and spirulina, a microalga that serves as food for the *Daphnia*). When carrying out the test, the ephippia were kept for 72 h in an incubator (ERI 180 DBO, Equitec^®^ 9, Equitec, Barcelona, Spain) at a temperature between 20 and 22 °C and 6000 l× light. After 72 h, the ephippia hatched and were fed with spirulina and left for 2 h.

After this time, the following serial concentrations of MMC (0.0025, 0.025, 0.25, 2.5, 25 µg/mL), OCT (0.004, 0.04, 0.4, 4, 40 µg/mL), and the MMC-OCT combinations (0.00125–0.002, 0.0125–0.02, 0.125–0.2, 1.25–2, 12.5–20 µg/mL) were prepared using the Daphtoxkit culture medium while the pH was maintained between 7 and 7.5 through the addition of 0.1 M NaOH or HCl solutions. For each concentration, 5 replicates were performed by introducing 10 mL of solution and 5 neonates in each well. Negative controls were also performed by introducing 10 mL of culture medium with 5 neonates per well without added substances.

Once each test plate was prepared, they were kept in the dark at a temperature between 20 and 22 °C for 48 h. After this time, the *D. magna* individuals were counted depending on their degree of mobility with respect to the untreated controls. An individual *D. magna* was considered alive if it exhibited visible movement (such as swimming or twitching); it was considered dead if it was still and showed no response or was unable to swim after 15 s of mild agitation. Abnormal behavior of individuals was considered. After counting, the EC_50_ was determined, which indicated the concentration of the compound that caused 50% immobilization.

#### 3.9.2. *Aliivibrio fischeri* Bioluminescence Inhibition Test

An acute toxicity test with the marine bacterium *A. fischeri* was performed following the steps described in UNE-EN-ISO 11348-3 2009. First, a saline medium of 2% (*w*/*v*) concentration was prepared. This medium was used to prepare the following serial concentrations of MMC (0.0025, 0.025, 0.25, 2.5, 25 µg/mL), OCT (0.004, 0.04, 0.4, 4. 40 µg/mL) and the MMC-OCT combinations (0.00125–0.002, 0.0125–0.02, 0.125–0.2, 1.25–2, 12.5–20 µg/mL) being the pH of each solution adjusted between 7 and 7.5 with the addition of 0.1 M NaOH or HCl solutions, when necessary.

To prepare the bacteria for the assay, we used the freeze-dried bacteria distributed by Vidrafoc (Barcelona, Spain) using the BioFix^®^ kit. The vial of bacteria was rehydrated using the reactivating solution distributed by the supplier in the kit. First, 5 mL of solution was added to the vial, and after gentle shaking, it was placed in the refrigerator for 5 min. Then 6 mL of the same solution was added, and the bacterial suspension was stored in the fridge. In these assays, four replicates per concentration were performed, and a negative control, in which no test compound was added, was included. Twenty-one vials were placed in a thermostatic bath at 15 °C. 0.5 mL of the reactivated bacteria solution was placed in the vials and allowed to warm up for 10 min. Subsequently, the basal luminescence of the solution lacking bacteria was measured using a BioFix^®^ Lumi-10 luminometer (Macharey-Nagel, Düren, Germany), covering the 380–660 nm spectral range. Subsequently, 0.5 mL of the solutions under study was added, and the measurements were repeated after 30 min (UNE EN ISO 11348-3 (2009)). The data obtained was represented as the percentage of luminescence inhibition for each concentration compared to the control to obtain the EC_50_ (concentration that results in a 50% decrease in bioluminescence).

#### 3.9.3. Statistical Analysis of the Concentration Effect

Logistic logit regression was employed to determine the dose–response curves for both indicators. Using the XLSTAT software (version 2014.5.03), the corresponding EC_50_ and EC_10_ values, along with their 95% confidence intervals (CI), were calculated. The statistical validity of these dose–response models was subsequently verified using the chi-square test.

## 4. Conclusions

OCT and MMC show additive antimicrobial effects against clinical strains of MRSA and *E. coli* UTI. MMC was more effective against the pathogenic Gram-positive bacteria than against the Gram-negative bacteria, whereas the opposite trend was observed for OCT. Selected combinations of both antimicrobials effectively eliminated bacteria while only maintaining acceptable cytotoxicity toward eukaryotic cells at the lowest concentrations evaluated in 2D. Moreover, cytotoxicity was reduced in the 3D fibroblast spheroid model, with the lowest concentrations assayed showing minimal toxic effects. In addition, in the most challenging scenarios, the combination of both antimicrobials was able to avoid biofilm formation of both MRSA and *E. coli* UTI and eliminate intracellular MRSA infecting macrophages, although these findings are limited to the MRSA strain evaluated in this study and cannot be extrapolated to other intracellular bacterial pathogens. Regarding environmental safety, the ecotoxicological results showed a bioindicator-dependent response. In *A. fischeri*, the OCT-MMC combination showed lower toxicity than the individual compounds. However, in *D. magna*, the mixture displayed acute toxicity comparable to OCT alone, indicating that the combination did not reduce toxicity in this organism and that OCT was the main driver of the observed effect under the tested conditions. It shows that those already approved drugs in combination promote bacterial elimination in their different forms. These findings may suggest that the additive effect could potentially contribute to reducing resistance pressure due to the reduced doses used, expanding the antimicrobial arsenal without relying on antibiotics only. However, further studies are needed to guarantee the safe use of this combination at selected doses and to guarantee its non-systemic absorption after topical administration.

## Figures and Tables

**Table 1:** MICs and MBCs of MMC and OCT against MRSA and *E. coli* UTI strains.

		MRSA	*E. coli* UTI
MMC	MIC	0.1 µg/mL	0.75 µg/mL
	MBC	0.25 µg/mL	2 µg/mL
OCT	MIC	1–2 µg/mL	3–4 µg/mL
	MBC	25 µg/mL	4 µg/mL

**Table 2:** CE_50_ (µg/mL), CE_10_ (µg/mL) and their respective CI for OCT, MMC, and their combinations (OCT-MMC) on *A. fischeri* and *D. magna*.

	***A. fischeri***			
	**CE_50_** **(µg/mL)**	**CI** **(µg/mL)**	**CE_10_** **(µg/mL)**	**CI** **(µg/mL)**
OCT	0.063	0.031–0.113	0	0–0
MMC	0.303	0.214–0.436	0	0–0
OCT-MMC	2.082	1.155–3.995	0	0–0
	***D. magna***			
	**CE_50_** **(µg/mL)**	**CI** **(µg/mL)**	**CE_10_** **(µg/mL)**	**CI** **(µg/mL)**
OCT	0.017	0.004–0.045	0	0–0.001
MMC	14.941	5.602–86.041 *	0.349	0.040–1.018
OCT-MMC	0.010	0.001–0.030	0	0–0.001

* EC50 estimate based on extrapolation beyond the highest tested concentration.

## Data Availability

Data will be made available on request.
